# Supplementary material for: Dietary Phytic Acid, Dephytinization, and Phytase Supplementation Alter Trace Element Bioavailability—A Narrative Review of Human Interventions
Source: Nutrients. 2024 Nov 27;16(23):4069. doi: 10.3390/nu16234069 (PMC11643945; doi:10.3390/nu16234069)
Supplement: Supplementary file 1 [file nutrients-16-04069-s001.zip › nutrients-3334669-supplementary.pdf]

Evidence Table S1: Intervention studies exploring the impact of phytase on micronutrient absorption							
Ref	Aim / Design	Phytase Intervention		Study population	Country/ Setting	Outcome measures	Main findings
		**Type**, **Dose** & **Duration**	Test meal/ supplement provided with phytase				
**[1]**	**Aim:** To investigate ways to increase Fe absorption from FeSO4-fortified teff injera in normal-weight healthy women.<br><br>**Design:** Crossover interventional study	**Type:**<br>*Aspergillus* niger<br><br>**Dose:**<br>380 FTU<br><br>**Duration:**<br>3 d	100% Teff Flour (Traditional Fermentation):<br>PA Content: 0.63 ± 0.15 g/100g dm<br><br>90% Teff Flour + 10% Whole Wheat Flour:<br>PA Content: 0.24 ± 0.01 g/100g dm<br><br>75% Teff Flour + 25% Whole Wheat Flour:<br>PA Content: 0.01 ± 0.01 g/100g dm<br><br>100% Teff Flour + Aspergillus niger Phytase:<br>PA Content: 0.02 ± 0.01 g/100g dm<br>PA Reduction: 98%	N = 17<br><br>Healthy Adults	**Country:**<br>Zurich, Switzerland<br><br>**Setting:**<br>University study	Fe absorption	No significant difference in Fe absorption between injera with 10% whole wheat flour and injera with added phytase (P > 0.05)<br><br>No significant increase in Fe absorption compared to 100% teff flour injera (P > 0.05)
**[2]**	**Aim:** Investigate the effect of adding exogenous phytase to SQ-LNS on Zn	**Type:**<br>*Aspergillus* Niger	Breakfast and lunch meals of a millet-based porridge	N = 26	**Country:**<br>Gambia	AGP<br><br>TDZ	Phytase supplementation increased both FAZ (greater among girls than

	absorption in young children.<br><br>**Design:** RCT	**Dose:**<br>588 x 2 FTUs<br><br>**Duration:**<br>1 d for 2 meals (breakfast and lunch)	(20 g SQ-LNS, lipid-based nutrient supplement, containing 8 mg Zn)	Healthy Children (18-30 mo of age)	**Setting:**<br>Medical Research Council of Gambia	FAZ<br><br>TAZ	boys and greater among breastfed children than non-breastfed children) and TAZ significantly (*P* < 0.001).<br><br>Dietary Zn intake from test meals (dependent on the quantity of porridge consumed) was inversely associated with FAZ (*P* < 0.01).<br><br>AGP modified the effect of exogenous phytase added to SQ-LNS on the total absorption of Zn (*P* for interaction = 0.04)
**[3]**	**Aim:** To assess the effect on Fe absorption of a lipid emulsion given 20 minutes before or together with an Fe-fortified maize meal.<br><br>To assess Fe absorption from a micronutrient powder (MNP) given with a nutrient-dense RUTF (Ready-to-Use-Therapeutic Foods) and/or a microbial phytase.	**Type:**<br>*Aspergillus* niger<br><br>**Dose:**<br>190 FTUs<br><br>**Duration:**<br>2 d with 15 d off between	4 different meals based on whole maize, with a 14-d interval (d 1, 15, 29, and 43), fortified with an MNP given with the following:<br>i) an RUTF<br>ii)phytase<br>iii) both an RUTF and phytase or<br>iv) neither	N = 41<br><br>Healthy Adults	**Country:**<br>Switzerland<br><br>**Setting:**<br>University	Fe absorption	The addition of a microbial phytase increases Fe absorption from FeSO4, with and without the addition of RUTF.<br><br>The combination of an RUTF and phytase has no additional effect on Fe absorption.

	**Design:** RCT						
**[4]**	**Aim:** To investigate the effect of adding phytase to millet-based porridge on Zn absorption in young children.<br><br>**Design:** RCT	**Type:** *Aspergillus* niger<br><br>**Dose:** 20.5 FTU<br><br>**Duration:** 1 d	Millet - based complementary food fortified with Zn alone or with Zn and the enzyme phytase added immediately before consumption.	N = 35<br><br>Healthy Children (12-24 mo of age)	**Country:** Ouagadougou, Burkina Faso<br><br>**Setting:** University Hospital Yalgado Ouédraogo	FAZ<br><br>PZn	FAZ increased significantly from 9.5 ± 3.4% to 16.0 ± 5.1% (P < 0.0001) with added phytase.<br><br>EZP measured as 3.6 ± 0.5 mg/kg; no correlation with FAZ observed for either test meal.<br><br>Adding phytase to Zn-fortified cereal porridge improves Zn absorption in children.
**[5]**	**Aim:** To optimise Zn absorption from maize and sorghum porridges fortified with Zn by evaluating the effects of PA and polyphenols as inhibitors, and EDTA and phytase as enhancers.<br><br>**Design:** RCT crossover single-blind	**Type:** *Aspergillus* niger<br><br>**Dose:** 190 FTUs phytase<br><br>**Duration:** 1 d	3 test meals were served in a crossover design and randomly.<br><br>Meal A: Maize porridge<br>Meal B: Maize porridge+phytase<br>Meal C: Maize porridge, dephytinised<br><br>Meals B + C were administered, each after a 4-wk washout period on d 29 and 57,<br>respectively.	N = 60<br><br>Healthy Adults	**Country:** Switzerland<br><br>**Setting:** Clinical Trials Center of the University Hospital Zurich	FAZ<br><br>PZn	The dephytinisation of the meal during preparation and the addition of the phytase immediately before consumption both significantly increased FAZ by 80% (P<0.001 for both).<br><br>Consuming the active phytase together with the cereal meal increased Zn absorption to the same extent as when the meal

							was completely dephytinised before consumption.
**[6]**	**Aim:** To optimise Fe bioavailability from a lipid-based nutrient supplement (LNS) named complementary food fortificant (CFF) in young children by evaluating the effects of AA, NaFeEDTA, and phytase on Fe absorption.<br><br>**Design:** RCT crossover design	**Type:**<br>Microbial phytase *Aspergillus* niger<br><br>**Dose:**<br>200 mg/portion ~400 FTU<br><br>**Duration:**<br>1 d	3 different test meals (A, B, and C) on 3 consecutive d were fed to each child.<br><br>Meal A: millet porridge<br>Meal B: millet porridge + phytase<br>Meal C: millet porridge + phytase + AA	N = 18<br><br>Healthy Children (19-36 mo of age)	**Country:** Natitingou<br><br>**Setting:** Benin hospital	Fe absorption	Phytase almost doubled the fractional Fe absorption from FeSO4-fortified test meals ($P < 0.001$).<br><br>When adding phytase and extra AA, the total absorbed Fe was more than doubled. Fe absorption when adding phytase plus extra AA was higher than adding only extra AA without phytase.
**[7]**	**Aim:** To test the efficacy of a low-Fe and low-Zn micronutrient powder (MNP), containing Fe as NaFeEDTA, AA, and an exogenous phytase active at gut pH, in improving Fe and Zn status and promoting somatic growth in South African school children with low Fe status.	**Type:**<br>*Aspergillus* niger<br><br>**Dose:**<br>380 FTU<br><br>**Duration:**<br>5 d/wk for 23 wk; for a total of 113 feeding d.	Daily bowl of 250 g (wet weight) sweetened maize porridge.	N = 189<br><br>Healthy Children (school-aged)	**Country:** Kimberley, Northern Cape, South Africa<br><br>**Setting:**<br>2 primary schools	Body Fe<br><br>Serum Zn	MNP decreased Fe deficiency by 75% compared with 35% in the control group and Zn deficiency by 36% compared with 9%.<br><br>Body Fe doubled from 1.8 to 3.6 mg/kg in the treatment group and increased from 2.0 to 2.8 mg/kg in the control group.

	**Design:** Double blind placebo-controlled trial						Half of the children in the study were mildly Zn deficient at baseline and provision of the MNP significantly reduced the prevalence of Zn deficiency by 66%.
**[8]**	**Aim:** To optimise Fe absorption from a low-Fe micronutrient powder intended for in-home fortification of complementary foods by evaluating combinations of NaFeEDTA, AA, and a microbial phytase active at gut pH.<br><br>**Design:** Crossover Interventional Studies (6 separate Fe absorption studies)<br><br>*Study 1*: Fe absorption evaluation from NaFeEDTA fortified meals without any enhancers.<br><br>*Study 2:* Comparison of Fe absorption from $FeSO_4$ fortified meals with and	**Type:** *Aspergillus* niger<br><br>**Dose:** 190 FTU<br><br>**Duration:** 2 d	Maize porridge fortified with a micronutrient powder containing either ferrous sulphate or NaFeEDTA<br><br>Supplement: whole-maize porridge fortified with 3 mg stable isotope–labelled FeSO4 or NaFeEDTA	N = 101<br><br>Healthy Adults	**Country:** Switzerland<br><br>**Setting:** University of Zurich	Fe absorption	The addition of phytase when Fe was present as either NaFeEDTA or FeSO4 significantly increased Fe absorption.<br><br>The addition of phytase when Fe was present as NaFeEDTA and the meal containing AA also significantly increased Fe absorption.

	without the addition of phytase.<br><br>*Study 3*: Assessment of the impact of AA on Fe absorption from NaFeEDTA fortified meals.<br><br>*Study 4:* Examination of the combined effect of phytase and AA on Fe absorption from NaFeEDTA fortified meals.<br><br>*Study 5:* Testing the efficacy of L-α-glycerophosphocholine as an enhancer of Fe absorption from $FeSO_4$ fortified meals.<br><br>*Study 6:* Investigation of Fe absorption from meals containing combinations of NaFeEDTA, AA and phytase.						

**[9]**	**Aim:** To investigate whether the addition of phytase to the wheat bread could mitigate any potential negative impact on Fe status due to the presence of dietary fibre and PA.<br><br>**Design:** RCT<br><br>300 g of fibre-rich wheat bread as a substitute for part of their habitual diet for a period of 16 wk and received either bread prepared without phytase (wheat bran bread group) or the same bread prepared with phytase (wheat bran bread + phytase group).	**Type:**<br>*Aspergillus* niger phytase produced from *Aspergillus* Oryzae NOVO-L<br><br>**Dose:**<br>2500 PTU/100 g<br><br>**Duration:** 16 wk	All wheat bread: 32 % white wheat flour, 11 % whole wheat flour, 8 % wheat bran (particle size: 1.2 mm – 0.6 mm), 3% specially formulated flour, 1.6 % sugar, 1.1 % salt, 1.9 % yeast, 1.8 % sunflower oil, 40 % water.	N = 41<br><br>Healthy Adults	**Country:**<br>Denmark<br><br>**Setting:**<br>At home meals + lab measurements	Serum ferritin levels	The consumption of the recommended daily intake of fibre-rich wheat bread led to a reduction in Fe status among women with initially sufficient Fe stores.<br><br>The addition of phytase to the wheat bread did not effectively prevent this decline in Fe status over the 4 mo intervention period.
**[10]**	**Aim:** To assess the inhibitory effects of PA and polyphenols on Fe absorption from ferrochel.<br><br>**Design:** Controlled experimental study<br><br>Different meals were administered to participants to evaluate Fe	**Type:**<br>Phytase from wheat<br><br>**Dose:**<br>304U<br><br>**Duration:**<br>1 d	A) Basal breakfast: 3 mg Fe as ferrous sulphate (labelled with 37 kBq 55Fe)<br>B) Basal breakfast + 3 mg Fe as ferrochel labelled with 59Fe<br>C) Basal breakfast + 3 mg Fe as ferrochel labelled with 59Fe + phytase<br>D) Basal breakfast + 3 mg Fe as ferrochel labelled with 59Fe + phytase	N = 74<br><br>Healthy Adults	**Country:**<br>Valencia (Carabobo State, Venezuela)<br><br>**Setting:**<br>NS	Fe absorption	Fe absorption from the basal breakfast prepared from precooked corn flour, enriched with Fe in test A and the same enriched meal administered with phytase in Test C was 50% greater when phytase was added.

	absorption under various conditions.						Absorption from the basal breakfast enriched with ferrochel in test B and with ferrochel phytase in test D was 61% greater in the presence of phytase.
**[11]**	**Aim:** To determine whether the addition of microbial phytase could enhance Fe absorption by degrading PA in the meal before it reaches the intestinal site of absorption.<br><br>**Design:** Controlled experimental study<br><br>*Study 1:*<br>Meals: Subjects consumed meals containing white wheat rolls supplemented with wheat bran, either with or without cereal phytase activity.<br>Fe Tracers: 2 different radio Fe tracers, 55Fe and 59Fe, were used to compare Fe absorption.	**Type:**<br>*Aspergillus* Niger<br><br>**Dose:**<br>Finase S40 contains a declared phytase activity of 4 x 107 PU/L. 5 mL of Finase was added to the test meal<br><br>**Duration:**<br>2 d	2 wheat rolls<br>(each prepared from 40 g unfortified white flour, (60% extraction) yeast, sugar, table salt and water).<br>The flour was fortified with 3.7 mg Fe as FeSO4 per 80 g flour.<br>Native Fe content was 0.4 mg.<br>2 rolls were served with 20 g of margarine and 150 mL water.	N = 20<br><br>Healthy Adults	**Country:**<br>Sweden<br><br>**Setting:**<br>NS	Fe absorption	No significant differences in Fe absorption were found between meals containing wheat bran with or without cereal phytase activity.<br><br>Addition of microbial phytase from Aspergillus niger to the meal with phytase-deactivated wheat bran significantly increased Fe absorption from 14.3 ± 2.6% to 26.1 ± 3.8% ($P < 0.0001$).<br><br>The high activity of microbial phytase at physiological pH conditions suggested effective and complete degradation of PA in the stomach when A. niger

	Study 2:<br>Meals: Participants consumed meals containing phytase-deactivated wheat bran, either with or without the addition of microbial phytase from Aspergillus niger.<br>Fe Tracers: 2 different radio Fe tracers, 55Fe and 59Fe, were used to compare Fe absorption.<br><br>The 2 types of rolls (A and B) in each study were given on alternate mornings after an overnight fast on 4 consecutive d in the order ABBA or BAAB.<br>The phytase used in Study 2 was added dropwise to the rolls before margarine spreading and without getting in contact with the bran before roll consumption.						phytase was given with the meal.
**Abbreviations:** PA: Phytic Acid, NS: Not specified, Fe: Iron, Zn: Zinc, MNP: Micronutrient powders, RUTF: Ready-to-Use-Therapeutic Foods, U: Units, FTU or PTU: Phytase units, D: day, Wk: weeks, Mo: months, AGP: α-1-acid glycoprotein, TDZ: Total dietary Zn, FAZ: Fractional absorption of Zn, TAZ: Total absorption of Zn, PZn: Plasma Zn, LNS: Lipid-based nutrient supplement, CFF: Complementary food fortificant, AA: Ascorbic acid							

Evidence Table S2: Intervention studies exploring the impact of phytic acid on micronutrient absorption						
Ref	Aim / Design	Intervention	Study population	Country / Setting	Outcome measures	Main findings
**[12]**	**Aim:** To determine the short-term effects of consuming low–PA maize on Zn absorption.<br><br>**Design:** Crossover interventional study (volunteers served as their own control subjects)	Polenta maize was fed for 3 meals/d and nothing else, for 2 d.<br><br>Group A: control maize on d 1 (70Zn added) and low–PA maize on d 2.<br><br>Group B: low–PA maize on d 1 and control maize on d 2 (67Zn added).<br><br>Control maize: maize polenta from a sibling isohybrid homozygous wild-type maize with a "normal" PA content.<br><br>LP maize: maize polenta from maize homozygous for the recessive LP 1-1; 17).	N = 5<br><br>Healthy Adults	**Country:** Denver (metropolitan area)<br><br>**Setting:** NS	Zn content<br><br>Zn absorption	Each subject had a consistently greater fractional absorption of Zn from the polenta prepared with the low–PA maize than from the control maize. The mean fractional absorptions of Zn from the low–PA and control maizes were 0.30 ± 0.13 and 0.17 ± 0.11, respectively.<br><br>The mean difference between the low–PA and control maizes was 0.13 ± 0.05 ($P < 0.005$). On average, the fractional absorption of Zn from polenta prepared from the low–PA maize was 78% greater than that from the polenta prepared with the control maize.
**[13]**	**Aim:** We investigated whether regular consumption of PA dampens its negative effect on nonheme-Fe absorption, as assessed by the serum Fe curve.<br><br>**Design:** Interventional study	8-wk dietary intervention in which each subject consumed either HP or LP diets.<br><br>The HP group received whole grain ready-to-eat cereals, whole wheat pasta/spaghetti, tortillas, bagels, bread and dinner rolls, corn tortillas,	N = 28<br><br>Healthy Adults	**Country:** Iowa, USA<br><br>**Setting:** University study, lab measurements	Fe absorption<br><br>Plasma hepcidin<br><br>Serum Fe	Plasma hepcidin concentration was higher in the LP group than in the HP group by 2.1 mg/L at baseline and 2.2 mg/L at post intervention ($P < 0.05$).

		brown rice, canned black beans, edamame, and tofu and was encouraged to consume generous amounts of nuts and other legume products high in PA.<br><br>The LP group received similar foods made from refined wheat and white rice, eggs, and cheese and was instructed to avoid HP foods.		+ at home meals		In the HP group, the AUC (area under the curve) increased by 41% compared to baseline ($P<0.0001$).<br><br>Baseline Fe absorption, as assessed by the serum Fe curve, was significantly lower ($P < 0.0001$) in the LP group than in the HP group even though subjects were randomly assigned to the groups. At post intervention, absorption was higher in the HP group than in the LP group after controlling for the baseline serum Fe response and hepcidin concentration ($P < 0.0001$), resulting in a 64% lower AUC in the LP group.
**[14]**	**Aim:** To evaluate the effect of PA on Mg absorption in adult human**s.**<br><br>**Design:** RCT	All test meals were based on 200 g PA-free white-wheat bread.<br><br>2 test meals (A: added PA and Mg, and B: no added PA) were randomly allocated to be served on d 1 or d 3.<br><br>D 1 and d 3: Test meals were divided into 2 identical portions and served at breakfast (07:30–08:30) and lunch (12:00–13:00) on the same d.	N = 20<br><br>Healthy Adults	**Country:** Switzerland<br><br>**Setting:** lab measurements + at home meals	Mg absorption<br><br>PA content	Addition of 1.49 mmol V to 200 g PA–free bread inhibited apparent Mg absorption significantly: 32.5 6.9% (test meal A) compared with 13.0 6.9% (test meal B) ($P< 0.0005$).<br><br>The inhibitory effect of PA on Mg absorption was dose dependent ($P<0.005$, unpaired Student's t test).<br><br>On the basis of a linear mixed model, a statistically significant effect of meal (with or without added PA; $P<0.001$) on Mg absorption was observed.

						The study-by-meal interaction was statistically significant (P<0.005), which indicated that Mg absorption was significantly influenced by the amount of PA added to the meal.
**[15]**	**Aim:** To determine the amount of different inositol phosphates in bread prepared in different ways and to relate these amounts to the bioavailability of Fe in humans.<br>**Design:** Crossover interventional study (each subject was its own control)	Test and control rolls were served on alternate mornings after an overnight fast on 4 consecutive d. Fe (ferrous sulphate) was added to the dough of all rolls except the bran rolls, to obtain a similar Fe content in all rolls.<br>5 experiments: the rolls in each experiment had different flours and different PA contents.<br>Test rolls:<br>*Exp 1*: whole rye flour + low extraction (55%) wheat flour.<br>*Exp 2*: whole wheat flour + low extraction (55%) wheat flour.<br>*Exp 3*: wheat flour (85% extraction).<br>*Exp 4:* whole rye flour + 85% extraction wheat flour.<br>*Exp 5*: wheat bran + low extraction (55%) wheat flour.	N = 49<br>Healthy Adults	**Country:** Sweden<br>**Setting:** University study	Fe absorption	In Experiment 2, Fe absorption from the whole wheat rolls was lower than from the control rolls. The difference was statistically significant (absorption ratio 0.79; P<0.05). In Experiments 3-5 the differences between Fe absorption from the test rolls and the control rolls were all statistically significant. The mean individual Fe absorption ratios (test rolls:control rolls) were 0.39 (P< 0.01), 0.32 (P<0.01) and 0.13 (P<0.01), respectively.

[16]	**Aim:** To examine whether intestinal adaptation results from a long-term high PA intake.<br><br>**Design:** Crossover interventional study	The effect of bran on Fe absorption was studied by comparing Fe absorption from wheat rolls, both with (A) and without (B) bran, in the same subject. The 2 types of rolls were served on alternate mornings after an overnight fast on 4 consecutive d in the order ABBA or BAAB. 2 A or B rolls were served each morning. The A and B rolls were labelled with 2 different Fe isotopes. 2 wk after the last rolls were served, a blood sample was taken to determine the relative absorption of 55Fe and 59Fe.<br><br>Because of the higher Fe content of the bran (3.8 mg/portion), the Fe content in the wheat rolls was adjusted to 3.8 mg by adding ferrous sulphate to the dough.	N = 13<br><br>Healthy Adults	**Country:** Sweden<br><br>**Setting:** NS	Relative absorption of Fe<br><br>Fe content<br><br>PA content	The difference in Fe absorption from wheat rolls and bran rolls between the 2 groups was not statistically significant. The inhibition of the Fe absorption by substituting part of the wheat flour by wheat bran was the same in both groups as shown by the absorption-ratio values.
[17]	**Aim:** The current study was designed to answer the questions: (1) does delivery method of PA during a meal change cystatin SN measurement and (2) does habitual pre-study PA intake.	Group 1: 500 mg PA powder sprinkled on the meal.<br><br>Group 2: 500 mg PA given as capsules with the meal.<br><br>Group 3: no PA supplement.<br><br>2 separate blood samples were collected pre-meal. After the	N = 30<br><br>Healthy Adults	**Country:** USA<br><br>**Setting:** School of Nursing, Wichita State University,	Serum Fe<br><br>Total salivary cystatin SN<br><br>Pre-meal cystatin SN, post-meal	Variation in pre-to-post meal cystatin SN accounted for 40% of Fe recovery in group 1 ($p<0.04$), but not groups 2 or 3.<br><br>Significant correlations were noted between the % max Fe recovery and mean dietary PA intake in groups 1 and 3 (p1⁄4 .03, $R2=0.76$ and $p=0.003$,

	**Design:** Single-blinded interventional study.	initial blood draw, a challenge meal was administered.<br><br>Dietary analysis.		Wichita, KS, USA	cystatin SN and pre-to-post meal cystatin SN (cystatin SN recovery)<br><br>Max Fe recovery	R2=0.7, respectively), but not group 2.<br><br>Pre-meal, and cystatin SN recovery were significantly negatively and positively associated with % max Fe recovery among all participants, respectively ($p<0.001$, model $R^2=0.91$).<br><br>When typical PA intake by group were accounted for in model 2, there were significant differences in % max Fe recovery between groups 1 and 2, and groups 2 and 3 ($p<0.0001$).<br><br>In correlation analysis, pre-to-post meal cystatin SN, and typical PA intake were significantly and positively correlated with improved % max Fe recovery in all subjects ($R^2=0.49$, 0.82, $p<0.0001$, =0.004, respectively), and within group 1 ($R^2=0.69$, 0.83, $p=0.02$, 0.001, respectively).<br><br>Group 3 % max Fe recovery was positively correlated with pre-participation PA intake ($R^2=0.82$, $p=0.001$).

**[18]**	**Aim:** To evaluate the effect of dephytinisation of a complementary food based on wheat and soy on Zn and Cu apparent absorption in healthy adults.<br><br>**Design:** RCT with crossover design	1st test meal: randomly allocated, complementary food with the native PA concentration or dephytinised complementary food.<br><br>Test meals: mixing 40 g dry complementary food (native PA or dephytinised) + 5 g sugar + 300 g hot water, labelled extrinsically with stable isotope solutions (0.4 mg 70Zn and 0.5 mg 65Cu).<br><br>D 1: Weight, height, blood sample.<br><br>D 2: After overnight fast, labelled test meals were administered in the morning and again 4 h later. No food or drink was allowed between test meals and 4 h after intake of the 2nd test meal. A standardised dinner and drinking water were provided on d 2.<br><br>Wash-out between the 2 study periods: 2–4 wk.	N = 10<br><br>Healthy Adults	**Country:** Zurich, Switzerland<br><br>**Setting:** NS	PA concentrati on<br><br>Cu absorption<br><br>FAZ	Apparent fractional Zn absorption was significantly (P 0.005) lower from the complementary food with native PA concentration (22.8 8.8%) compared with the dephytinised complementary food (34.6 8.0%).<br><br>No effect was observed on Cu apparent absorption, and these results thus add to the body of evidence that PA is not an inhibitor of Cu absorption.
**[19]**	**Aim:** To study the dose-dependent effect of adding different amounts of sodium PA to a single meal and to determine the whole-body	Test meals consisting of white wheat rolls to which known amounts of sodium PA (25–250 mg PA) were added immediately before serving. 4	N = 40<br><br>Healthy Adults	**Country:** Sweden<br><br>**Setting:** NS	Zn absorption<br><br>Ca retention	A decrease in Zn absorption (p<0.001) was seen in the interval of 0–100 mg PA (0–538 mmol PA).

	retention of Zn and Ca with radionuclide technique.<br><br>**Design:** Interventional study	groups of 10 subjects ate test meals containing each level of PA. All subjects also received a meal to which no sodium PA was added. The groups were served the meals in the following, not randomised, order: the 1st one 0 and 250 mg, the 2nd 100, 50 and 0 mg, the 3rd 75, 140 and 0 mg, the 4th 25, 175 and 0 mg PA.<br><br>Subjects were served 2 or 3 meals each after an overnight fast.<br><br>Totally 105 meals were served, 36 meals in which no PA was added and 9–10 meals on each level of PA (25-250 mg).<br><br>The activities of 65Zn and 47Ca were then measured by whole-body counting 3-6 times over a 4-wk period after each meal.				Even in the interval of 100–250 mg PA (538–1344 mmol PA) the decrease in Zn absorption was significant ($p<0.05$).<br><br>A decrease in Zn absorption, calculated with log- transformed values ($p=0.01$) when the meal was added 50 mg PA (269 mmol PA) compared to the meal with no added PA.<br><br>The molar ratios of PA: Zn in the meals were 0.0, 2.9, 5.7, 8.6, 11.5, 16.0, 20.0 and 28.6:1. By the molar ratio PA:Zn of 5.7:1 the Zn absorption was decreased ($p=0.01$).<br><br>Decrease ($p<0.001$) was seen in the interval of 0–250 mg PA (0–1344 mmol PA).<br><br>The Ca retention at d 7, calculated with log- transformed values, was decreased ($p=0.03$) when 100 mg PA (538 mmol PA) was added to the meal, compared to the meal with no added PA.<br><br>The molar ratios of PA: Ca in the meals were 0.0, 0.02, 0.04, 0.06, 0.08, 0.11, 0.14 and 0.20:1. By the molar ratio PA:Ca of 0.08:1, Ca absorption was decreased ($p=0.03$).

						The correlation coefficient (r) between Zn absorption and Ca retention at d 7 in the meals with no added sodium PA was 0.484 ($p<0.05$).
**[20]**	**Aim:** To determine the effect of dietary PA on the quantity of Zn absorbed, with special attention to late pregnancy and early lactation.<br><br>**Design:** RCT	Study of Zn absorption at 8 (phase 1) and 34 (phase 2) wk of gestation and 2 (phase 3) and 6 (phase 4) mo of lactation.<br><br>Randomly assigned to 2 ad libitum feeding groups: LP maize (LP; 1.6 mg/g) or control maize (C; 7.1 mg/g).<br><br>On the d of Zn stable isotope administration, participants cooked and consumed all their meals in their home in the presence of a field research assistant. Intakes of each food were accurately measured at each of 3 meals and a duplicate diet sample prepared accordingly.	N = 22<br><br>Healthy Pregnant Women	**Country:** Rural township of San Juan Comalapa, Department of Chimaltenango, in the Western Highlands of Guatemala<br><br>**Setting:** at home + lab measurements	TAZ<br><br>FAZ<br><br>Total Zn concentration	Dietary Zn intake was comparable between groups, but the LP group had significantly lower PA intake.<br><br>The LP group had a more favourable PA-to-Zn molar ratio, suggesting better Zn absorption.<br><br>TAZ increased significantly from the first trimester to lactation in both groups, with a greater increase in the C group.<br><br>Early lactation TAZ was substantially higher than predicted for non-pregnant, non-lactating women, especially in the C group.<br><br>Zn absorption increases during late pregnancy and early lactation.
**[21]**	**Aim:** To investigate whether fractional Ca absorption from tortilla meals prepared from maize with approximately 60% PA reduction is significantly greater than from tortilla meals prepared from wild-type control maize.	Fractional Ca absorption from a test meal of tortillas prepared from a low-PA maize was compared with that from a tortilla test meal prepared from the isohybrid wild-type control maize. Test meals were	N = 5<br><br>Healthy Adults	**Country:** Colorado, USA<br><br>**Setting:** NS	Ca absorption	Mean fractional absorption of Ca from the low-PA maize tortilla meals was 0.50 ± 0.03 compared with a mean of 0.35 ± 0.07 from the tortilla meals prepared from the isohybrid wild-type control maize ($p< 0.01$).

	**Design:** Crossover interventional study	administered after an overnight fast at approximately 8am in the presence of one of the investigators. Wash-out period of 4 wk, subjects consumed the alternative test meal.<br><br>Urine sample.				
**[22]**	**Aim:** To investigate the PA effect on human Ca nutrition.<br><br>**Design:** RCT	Subjects were studied 3 times, 4 wk apart. Test meals were ingested as breakfast, after an overnight fast.<br><br>The high- and low-PA soybean sources were randomly assigned to the subjects at the 1st 2 test meals. Milk provided the Ca source at the 3rd meal.	N = 16<br><br>Healthy Adults	**Country:** Omaha, Nebraska<br><br>**Setting:** NS	Ca absorption<br><br>Fractional Ca absorption	Ca absorption from the low PA beans was greater than from the high PA beans in 15 of the 16 subjects.
**[23]**	**Aim:** To test the hypothesis that wholegrain rye bread with very low amounts of PA would improve markers of Fe status as compared to whole grain rye bread with high amounts of PA.<br><br>**Design:** RCT (double-blind, parallel-design intervention study)	Group 1: wholegrain rye bread made from blanched rye and thus containing the natural amount of PA (mean=77 mg/200 g bread). Group 2: dephytinised whole grain rye bread (mean<1.0 mg PA/200 g bread).<br><br>Both the low-PA and the high-PA bread (200 g) were alternately distributed to the subjects during this intervention.	N = 55<br><br>Healthy Adults	**Country:** Sweden<br><br>**Setting:** NS	S-ferritin<br><br>Body Fe reserves<br><br>Total dietary PAintake<br><br>Plasma alkylresorcinol concentrations	Of all the studied Fe biomarkers, the only observed changes were that S-ferritin (p<0.018) and amount of body Fe reserves (p<0.035) decreased within the low-PA bread group following 12 wk of intervention.<br><br>The low-PA bread group increased plasma alkylresorcinol concentrations from baseline (p<0.002).<br><br>In the low-PA bread group there was an inverse relationship between logarithmic transformed change in

		Each d for a period of 12 wk.			Total wholegrain intake	S-ferritin from baseline to post-intervention and total wholegrain intake (incl., wholegrain intake study bread) from mean meals and wholegrain intake from the study bread during the intervention (r=−0.415, p=0.044, and r=−0.478, p=0.018, respectively).
**[24]**	**Aim:** To test the inhibitory effect of dietary Ca, in Western diets with high and low PA content, on Zn absorption.<br><br>**Design:** 2 X 2 factorial design, interventional study.	Zn absorption was measured from 4 different 1-d menus, all of which were consumed by each subject in random order.<br><br>The menus contained 11.5 mg Zn, a moderate (700 mg/d) or high (1800 mg/d) amount of dietary Ca, and a low (440 mg/d) or high (1800 mg/d) amount of PA.<br><br>The menus: (a) Moderate-Ca, low-PA ,(b) Moderate-Ca, high-PA, (c) High-Ca, low-PA ,(d) High-Ca, high-PA.<br><br>4 wk between each menu.<br><br>The study lasted 16 wk, with absorption measurement every 4 wk. The subjects consumed a 1-d experimental menu for 2 consecutive d. On the 2nd d, the entire menu (3 meals) was	N = 10<br><br>Healthy Adults	**Country:** North Dakota, USA<br><br>**Setting:** Laboratory	Zn absorption	In healthy women consuming 1-d menus of ordinary foods (some fortified with Ca), dietary PA reduces Zn absorption, but Ca does not impair Zn absorption, regardless of whether dietary PA is low or high.<br><br>Fractional Zn absorption was significantly lower with high dietary PA.<br><br>Fractional Zn absorption averaged 10 percentage points lower with the high- than with the low PA diet.<br><br>Absolute Zn absorption was 25% lower with the high- than with the low-PA diet, an absorption difference of 1 mg/d.

		extrinsically labelled with 7.4 kBq (0.2 lCi) 65Zn tracer.				
**[25]**	**Aim:** The objective was to investigate whether either an extensive reduction in the PA content of infant cereals or the use of milk-based, Fe-fortified infant formula would improve Fe and Zn status in infants.<br><br>**Design:** Double-blinded RCT	Infants were randomly assigned to 3 cereal groups for 6 mo: (a) control commercial milk-based cereal and porridge, (b) PA-reduced milk-based cereal and PA-reduced porridge, (c) infant formula and porridge with the usual PA content.	N = 267<br><br>Healthy Infants<br><br>(6 - 12 mo)	**Country**: Sweden<br><br>**Setting**: 6 healthy-baby clinics in Umeå	Hb<br><br>Serum ferritin<br><br>Serum zinc	Extensive reduction in the PA content of weaning cereals had little long-term effect on the Fe and Zn status of infants.<br><br>Despite the reduced daily PA intake from infant cereals of ≤ 77%, there was no greater effect on Hb, serum ferritin, or Serum zinc than that with commercial weaning cereals (rich in PA).<br><br>Feeding infant formula, with lower Fe content but higher bioavailability, resulted in a significantly lower Hb and higher prevalence of anaemia than did feeding PA-reduced infant cereal.
**[26]**	**Aim:** The objective of study was to determine whether the long-term substitution of low-PA maize would enhance Zn absorption in a population that is dependent on maize as the major food staple.<br><br>**Design:** Community-level feeding RCT	Children (20 per group) were randomly assigned into 3 groups to be fed only the low-PA maize or 1 of 2 control maizes, the isohybrid wild-type maize or a locally grown maize, for a 10-wk period.	N = 60<br><br>Healthy Children<br><br>(6 –11 y)	**Country**: Guatemala<br><br>**Setting:** Village of Buena Vista, Guatemala	FAZ<br><br>TAZ	No significant differences in either the FAZ or TAZ were seen between the maize groups.<br><br>Under the conditions of the present study, Zn absorption was not increased by the long-term use of low-PA maize in children whose major dietary staple is maize.

**[27]**	**Aim:** The objective of the study was to test whether LP beans provide more bioavailable Fe than a BB (biofortified beans) variety when served as part of a composite meal in a bean-consuming population with low Fe status.<br><br>**Design:** RCT with crossover design	Dietary Fe absorption from LP, Fe-biofortified, and control beans (CB) (regular Fe and PA concentrations) was compared.<br><br>Each participant acted as their own control. Each of the bean cultivars was fed to subjects for 1 wk, and participants received meals containing different bean varieties during each period (LP, BB, and CB meal). The study was divided into 2-wk (2 bean varieties) and 1-wk (1 bean variety) feeding periods.<br><br>The selected subjects were randomly assigned to start in wk 1 of each feeding period with 1 of the 3 different bean meals. Each bean meal was served for 5 consecutive d, 2 X d.	N = 29<br><br>Healthy Adults	**Country:** Rwanda<br><br>**Setting:**<br><br>University of Rwanda	Fractional Fe absorption<br><br>Total Fe amount absorbed from test meals	Fractional Fe absorption from LP beans, BBs, and CBs did not significantly differ.<br><br>The total amount of Fe absorbed from LP beans and BBs was 421 mg and 431 mg, respectively, and did not significantly differ, but was >50% higher ($P < 0.005$) than from CBs (278 mg; 95% CI: 150, 499 mg).<br><br>The LP beans were hard to cook, and their consumption caused transient adverse digestive side effects in ;95% of participants. Gel electrophoresis analysis showed phytohemagglutinin L (PHA-L) residues in cooked LP beans.
**[28]**	**Aim:** The aim of the present study was to test the potential of the LP trait to improve Fe bioavailability from beans.<br><br>**Design:** RCT with crossover double meal design	Subjects consumed 4 different bean porridges: (a) white coated LP line low in Polyphenol compounds (PP) (LP-W), (b) brown coated LP line high in PP (LP-B), (c) parent wild-type beans with normal PA levels: 1 brown	N = 20<br><br>Healthy Adults	**Country:** Switzerland<br><br>**Setting:**University study	Fractional Fe absorption<br><br>Total Fe absorbed per meal	The mean fractional Fe absorption and total amount of Fe absorbed from the LP bean porridges were significantly greater than from the bean porridges made with the parent beans.<br><br>The total amount of Fe absorbed from the LP bean lines was up to

		coated high in PP (wt-B) and 1 white coated low in PP (wt-W).<br><br>Participants were randomly assigned to consume the high- or low-PP beans on d 1 or 2 or on d 17 or 18. If the participants received the LP bean at the first meal d of the study (d 1 or 17), they received the wild-type bean with comparable PP levels on the following d (d 2 or 18) or vice versa.				163% higher than from their parents with native PA content.

Abbreviations: PA: Phytic Acid, NS: Not specified, Fe: Iron, Zn: Zinc, Ca: Calcium, Cu: Copper, FTU or PTU: Phytase units, Exp: Experiment, D: day, Wk: weeks, Mo: months, HP: high PA, LP: low PA, TAZ: Total absorbed Zn, FAZ: Fractional absorption of Zn, CC: Commercial milk-based cereal, PR: PA-reduced milk-based cereal and PA-reduced porridge, AA: Ascorbic acid, IF: Infant formula, CB: Control beans, PP: Polyphenol compounds, Hb: Haemoglobin

Evidence Table S3: Intervention studies exploring the impact of food dephytinisation on micronutrient absorption.							
Ref	Aim / Design	Intervention		Study population	Country/ Setting	Outcome measures	Main findings
		Type, Dose & Duration	Test meal / supplement provided with phytase				
**[29]**	**Aim:** To determine the relative bioavailability of an Fe-rich bread containing teff flour (50% weight of dry ingredients) and to study whether the addition of the enzyme phytase further improved Fe bio-availability.<br><br>**Design:** RCT<br><br>5 groups: (a) Control bread (CB); (b) teff bread (TB); (c) TB + level 1 phytase (TB + P1); (d) TB + level 2 phytase (TB + P2); (e) A supplement containing 10 mg of ferrous sulphate.<br><br>Participants ate 3 or 4 slices (50 g/slice) of TB or CB, or took a Fe supplement.	**Type of Phytase:** NS<br><br>**Dose:** (i) 0.01 g 100 g-1 (P1) and (ii) 0.015 g 100 g-1 of flour (P2)<br><br>**Duration:** 1 d	**Type of meal:** Teff bread<br><br>TB was developed using 50% of teff flour. To improve the bioavailability of Fe from the TB, the enzyme phytase was added to bread, at 2 levels of concentration: (i) 0.010 g 100 g-1 (P1) and (ii) 0.015 g 100 g-1 of flour (P2).	N = 18<br><br>Healthy Adults	**Country:** UK<br><br>**Setting:** Manchester Metropolitan University, UK	Total Fe intake<br><br>Serum Fe	Total Fe intake from 3 or 4 slices of TB + P2 (8.4 mg) was statistically significantly higher than levels ingested from consuming similar proportions of CB (5.1 mg; $P < 0.001$).<br><br>Serum Fe levels declined with time after the consumption of all interventions, with the exception of Fe-supplement which increased serum Fe levels by 0.5 μM.

**[30]**	**Aim:** The aim of the present study was to evaluate the effect of dephytinisation of soybean protein isolate on the absorption of Fe, Zn, Cu, Ca and Mn in healthy infants fed soy formula.<br><br>**Design:** RCT crossover design<br><br>Each subject was studied once while receiving the dephytinised formula and once while receiving the regular soya formula. Each study consisted of a 10 d wash-out period followed by a 3 d period during which a metabolic balance was conducted.	**Type of phytase:** *Aspergillus* niger<br><br>**Dose:** NS<br><br>**Duration:** 1 d	**Type of meal:** The composition of the products was similar to a commercial infant formula, except for the use of dephytinised soya-bean protein isolate in production of the experimental formula.	N = 9<br><br>Healthy Infants (69-191 d of age)	**Country:** Iowa, USA<br><br>**Setting:** Lora N. Thomas Metabolism Ward (Department of Paediatrics, University of Iowa, Iowa City, IA, USA)	Zn absorption<br><br>Fe absorption	Zn absorption, measured by a stable isotope technique, was significantly greater from the dephytinised formula (mean value 22·6 %) than from the regular soya formula (mean value 16·7 %; P=0·03).<br><br>Apparent absorption of Fe, based on faecal excretion of the isotope, was significantly greater (P=0·001) than erythrocyte incorporation of Fe from both formulas.
**[31]**	**Aim:** Fe bioavailability from a cereal containing its native PA content was compared with the bioavailability from a similar cereal product in which PA had been 88% degraded.<br><br>**Design:** RCT<br><br>The infants were fed an Fe-fortified cereal product containing the native PA content for 2 wk before the study.<br><br>The wheat flour used for cereal B was the same as cereal A, but was dephytinised by the addition of phytase.<br><br>Infants were randomly assigned to start	**Type of phytase**: *Aspergillus* niger<br><br>**Dose:** NS<br><br>**Duration:** 2 d	**Type of meal:** Infant cereals composed primarily of white wheat flour (70% extraction) and skim milk powder. Each test meal consisted of 25 g infant cereal mixed with 100 g hot deionized distilled water.	N = 12<br><br>Healthy Infants<br><br>(21-39 wk of age)	**Country:** Paris, France<br><br>**Setting:** Health Center	Fe bioavailability	No difference in Fe bioavailability was observed in this study.<br><br>Dephytinisation of infant cereals containing a relatively low native PA content and high amounts of AA is thus unnecessary to ensure adequate bioavailability of Fe.

	with test meal A or B. 4 test meals labelled with stable isotopes were given on 4 consecutive d in the order ABAB or BABA after an overnight fast. Venous blood samples: d 1 (baseline) and 19 (14 d after intake of the last test meal).		Fortified with CaCO3 and a vitamin premix (with AA).				
**[32]**	**Aim:** The aim of the present study was to evaluate the effect of dephytinisation of soy protein isolate on Mn absorption in adults.<br><br>**Design**: RCT<br><br>Subjects were to start with test meal a or b, followed by the other test meal during the 2nd part of the study. Added in both formulas: Fe, Zn, and AA.<br><br>*Study 1*: formula 1 (soy isolate containing the native amount of PA) was compared with formula 2 (dephytinised).<br><br>*Study 2:* formula 1 was administered twice: the normal amount of AA (625 pmol/L, or I 10 mg/L) being compared with twice this amount.	**Type of phytase:** *Aspergillus* niger<br><br>**Dose:** NS<br><br>**Duration:** 1 d	**Type of meal:** Liquid infant formulas (ready-to-feed) based on soy isolate containing the native amount of PA (formula 1) or virtually free from PA prepared by the addition of phytase (formula 2) were produced for the studies.	N = 16<br><br>Healthy Adults	**Country:** Switzerland<br><br>**Setting:** NS	Mn absorption<br><br>AA content	The fractional Mn absorption was approximately doubled (mean absorption increased from 0.7% to 1.6%) by the dephytinisation of soy formula, whereas increasing the AA content of a PA-containing soy formula had no effect on Mn absorption in adults.
**[33]**	**Aim:** The objective was to measure the influence of PA degradation on Fe absorption from cereal porridges.<br><br>**Design:** RCT<br><br>An exogenous phytase was used to fully degrade PA during the manufacture of 9	**Type of phytase**: *Aspergillus* niger<br><br>**Dose:** NS<br><br>**Duration:**	**Type of meal:** 18 roller-dried cereal porridges were prepared: 9 with native PA concentrations	N = 78<br><br>Healthy Adults	**Country:** Switzerland<br><br>**Setting:** NS	Fe absorption<br><br>PA content	PA degradation with the phytase enzyme was very efficient, and PA was decreased to ≤ 0.002% in all dephytinised porridges except the dephytinised wheat-soy blend, which contained 0.02% PA

	roller-dried complementary foods based on rice, wheat, maize, oat, sorghum, and a wheat-soy blend.<br><br>Fe absorption from the PA-free and native PA porridges prepared with water or milk (wheat only) was measured in adult humans with an extrinsic-label radio Fe technique.<br><br>AA was added to some porridges.	2 d	and 9 after dephytinisation with phytase. These were made from flours of 8 different cereal grains and a blend of wheat and soy flour. The wheat-soy blend included 60% extraction wheat flour and defatted soybean flour. The cereal flours used were ground polished rice, 60% extraction wheat flour, partly degermed whole-white maize, dehulled oat flour (steamed to deactivate lipase and then roller-dried, flaked, and ground), and 4				(reduced from 0.3%).<br><br>Dephytinisation had no influence on Fe absorption of wheat porridge reconstituted with milk (absorption ratio: 1.26; $P > 0.05$).<br><br>Dephytinisation significantly increased absorption when the wheat porridge was prepared with water (absorption ratio: 3.48) but had no influence on Fe absorption when the porridge was prepared with milk (absorption ratio: 1.11; $P > 0.05$).

			different sorghum flours. The dephytinised porridges were prepared similarly.				
**[34]**	**Aim:** This study aimed at assessing the effect of PA degradation in fonio through a food-based approach and to quantify its effect on Fe bioavailability in humans.<br><br>**Design:** RCT Crossover Design<br><br>Subjects were given 2 Fe-fortified fonio porridges labelled with 57Fe or 58Fe, on 2 consecutive d.<br><br>D 1: meal test Group 1 labelled 58FeSO4 FFP<br><br>D 2: meal test Group 1 labelled 57FeSO4 FWFP<br><br>D 1: meal test Group 2 labelled 57FeSO4 FWFP<br><br>D 2: meal test Group 2 labelled 58FeSO4 FFP	**Type of phytase:** Whole grain wheat flour as native phytase source.<br><br>**Dose:** Adding 25% of whole wheat flour. 2.96 +-1.3 PU per g (whole wheat).<br><br>**Duration**: 1 d	**Type of meal:** The test meals consisted of 2 fonio porridges of 240 g: single non-dephytinised fonio flour porridge (FFP) and mixed dephytinised fonio-wheat porridge (FWFP, ratio 3:1, weight-to-weight). Fonio and wheat flours were made from whole grains. Fortified with: 57Fe or 58Fe labelled FeSO4.	N = 15<br><br>Healthy Adults	**Country:** Benin<br><br>**Setting**: Department of Nutrition and Food Sciences, University of Abomey-Calavi in Benin	Fe absorption	Geometric mean Fe absorption from FFP and FWFP meal was 2.6% and 8.3% respectively.<br><br>Fractional Fe absorption from FWFP was 3.2 times higher compared to FFP.<br><br>Dephytinisation of fonio porridge with intrinsic wheat phytase increased fractional Fe absorption 3.2 times.
**[35]**	**Aim:** The aim of the present study was to	**Type of**	**Type of meal:**	N=42	**Country:**	Fe	When the PA content was

	investigate the non-heme Fe absorption from an Fe-supplemented oat-based beverage and to assess the role and importance of citric acid addition, phytase treatment and supplementation with different Fe compounds.<br><br>**Design**: RCT<br><br>4 absorption trials. In each trial, a portion (240 g) of the test product (T) or a reference dose (R) was served to the subjects as breakfast after overnight fasting on 4 consecutive days in the order of TRRT.<br><br>Trial 1: Fe absorption from product A by supplementation as FeAC was examined.<br><br>Trial 2: Fe absorption from product B by citric acid addition and FeAC supplementation was examined.<br><br>Trial 3: the Fe absorption from product C by citric acid addition, phytase treatment and FeAC supplementation was examined.<br><br>Trial 4: the Fe absorption from product D by citric acid addition, phytase treatment and FePP supplementation was examined.	**phytase:** *Aspergillus* niger<br><br>**Dose:** NS<br><br>**Duration:** 2 d	Fe-supplemented oat-based beverages.<br><br>Members of each trial were given 1 of the studied 4 products (A, B, C, and D) supplemented with Fe (1.3 mg/portion), Zn, Ca, Se and P.	Healthy Adults	Sweden<br><br>**Setting:** University of Goteborg	absorption	decreased (in product C), the Fe absorption was increased still more to 10.7% in comparison with product B ($p = 0.003$).<br><br>Fe absorption was improved 2.7-fold in product C by the citric acid addition and phytase treatment as compared with the untreated oat beverage, product A.
**[36]**	**Aim:** The study objective was to improve Fe status of children in rural Malawi by using a community-based method to remove dietary PA from maize flour.	**Type of phytase:** commercia l phytase	**Type of meal:** Corn-plus-soy porridge served 5/d. The	N = 10<br><br>Healthy Children	**Country:** Malawi<br><br>**Setting:** Mpemba	Zn protoporp hyrin concentrat	After dietary PA reduction for 40 d, Fe status improved in 10 healthy Malawian children who habitually consume a high-PA,

	**Design:** Interventional study<br><br>The FFQ identified the 6 most commonly consumed foods: unrefined maize flour, red kidney beans, peanut flour, tomatoes, bananas, and Chinese cabbage. Each mother was provided with these 6 foods every 3 d.<br><br>After receiving the provided foods for 6 d, each child began the Zn metabolic study. On the 1st d the subjects came to the Mpemba Health Center, where they were fed 3 meals and 2 snacks prepared from the same 6 foods.<br><br>PA was removed from the maize flour by stirring 1 part flour in 4 parts water and adding phytase. Supplement: Zn-free multivitamin daily.	enzyme<br><br>**Dose:** approximately 1 g phytase/5 kg flour; 5000 U/g<br><br>**Duration**: 40 d	porridge was a mixture of 80% unrefined white corn flour and 20% soybean flour, with vegetable oil and sugar added. Water was added to form a 20% slurry. In addition to the porridge, a sugar-based drink and a fruit or nut snack were provided 1/d. For the PA-reduced diet, PA content reduction was by phytase.	(2–5 y)	Health Centre	ions<br><br>Fe status<br><br>Soluble transferrin receptor	maize-based diet.<br><br>Soluble transferrin receptor and Zn protoporphyrin concentrations decreased following consumption of the reduced PA diet, indicating an improvement in Fe status.
**[37]**	**Aim:** To evaluate the relative importance of PA and PolyPhenols (PP) in Fe absorption from common beans to gather information for plant breeders to develop beans with Fe optimised for bioavailability.<br><br>**Design:** RCT crossover design	**Type of phytase:** *Aspergillus* niger<br><br>**Dose:** For meal B in study 5	**Type of meal:** Beans with non-inhibitory reference meal (RM) consisting of a bread roll (80 g) made from yeast-	N = 97<br><br>Healthy Adults	**Country:** Singapore<br><br>**Setting:** University	Fe absorption	Removing both PA and PolyPhenol increased Fe absorption 2.6-fold.<br><br>Removing the hulls, and thus most of the PolyPhenol from beans prior to dephytinisation,

	6 randomised studies were conducted, with only studies 5 and 6 focusing on dephytinisation. Studies 4–6 investigated the inhibitory effects of polyphenols (PP) and PA on Fe absorption, either individually or combined.<br><br>*Study 4:* Examined Fe absorption with and without bean hulls to assess the influence of PP in the presence of PA.<br><br>*Study 5:* Compared Fe absorption between whole beans and dehulled, dephytinised beans to analyse the combined impact of PP and PA.<br><br>*Study 6:* Assessed Fe absorption in the absence of PA by comparing dephytinised beans with dephytinised, dehulled beans.<br><br>In all studies participants served as their own controls. Each subject received 2 test meals labelled with 57Fe or 58Fe, consumed on consecutive mornings after fasting.	and meal A and B in study 6, 100 PU was added to the bean slurry after the homogeniz ation and the slurry was held at 558C for 60 min to allow complete PA degradatio n.<br><br>**Duration:** 1 - 2 d	fermented wheat flour, honey (7 g), and coconut fat (3 g).<br><br>Study 5: Whole bean meal (meal A) and Dephytinised, dehulled bean meal (meal B).<br><br>Study 6: Dephytinised bean meal (meal A) and Dephytinised, dehulled bean meal (meal B).				doubled Fe absorption.<br><br>Dephytinisation in the presence of PolyPhenol did not increase Fe absorption. Removing most of the PolyPhenol in the presence of PA did not significantly change Fe absorption.<br><br>Dephytinisation after dehulling and removal of most of the PolyPhenol increased Fe absorption to 13.9%.<br><br>Dephytinisation in the absence of PolyPhenol increased Fe absorption ~3.4-fold.
**[38]**	**Aim**: A study on Zn absorption in young and elderly healthy human subjects, whose Zn and protein-energy status were carefully assessed.<br><br>**Design:** Interventional study	**Type of phytase:** NS<br><br>**Dose:** NS<br><br>**Duration:** 2 d	**Type of meal:** Soya milks (100 ml) fortified with 25 mg Zn	*Study 1:*<br><br>N = 20<br><br>Healthy Adults<br><br>*Study 2:*	**Country:** Switzerland<br><br>**Setting:** Nestlé Research Centre	*Study 1:*<br><br>Serum zinc<br><br>Serum total protein	Mean Serum zinc and albumin were significantly lower in the elderly subjects in both experiments.<br><br>Serum transthyretin was significantly lower in the elderly subjects in study 1 only, and

	Study 1: 2 soya milks differing only in PA content were tested.<br><br>*Study 2:* Study 2 was undertaken when the results of study 1 showed that the natural PA content of formula A was too high for the purposes of the test. The protocol was identical to that of study 1 in every aspect, except that the soya milk with the natural PA content (0·26 g/200 ml) was replaced by a mixture of 100 ml of this milk and 100 ml of the dephytinised milk by a commercial phytase (50 % reduction in natural content: 0.13 g/200ml).<br><br>In both studies, 2 serum concentration curve (SCC) tests were conducted on each participant with a 3-wk interval between tests.			N = 19<br><br>Healthy Adults		Albumin<br><br>Transthyretin<br><br>PA content<br><br>*Study 2:*<br><br>Serum zinc<br><br>Serum total protein<br><br>Zn	serum total protein was lower in the elderly subjects in study 2 only.<br><br>PA significantly reduced the mean AUC 0–180 values in studies 1 and 2.<br><br>The natural PA content of the soya milk (0·26 g/200 ml) dramatically reduced absorption of a dose of 50 mg Zn, to a level undetectable using this test (study 1).
**[39]**	**Aim:** We evaluated the effect of PA and AA on fractional Fe incorporation into red blood cells in healthy infants fed soy formula by using a recently developed double stable isotope technique.<br><br>**Design:** Interventional study<br><br>3 separate studies were made with 10 infants in each study.<br><br>*Study 1:* compared product A with product B.<br><br>*Study 2:* compared product C with product D.	**Type of phytase:** FinaseS40<br><br>**Dose:** 40 PTU/L<br><br>**Duration:** 2 d	**Type of meal:** 4 soy formulas (A-D):<br><br>2 parallel products (A and B, C and D) were made, 1 containing the native PA content (A and C) and the identical product being	N = 10<br><br>Healthy Infants	**Country:** France<br><br>**Setting:** Children's Health Center	Fe absorption<br><br>PA<br><br>Serum ferritin<br><br>Geometric mean fractional incorporation	A statistically significant effect of PA removal was found in studies 1 and 2, with a greater increase of the Fe incorporation when 100% PA was removed (study 2) compared with 83% removal (study 1).<br><br>The geometric mean fractional incorporation increased from 5.5 to 6.8% ($p < 0.05$; study 1) and from 3.9 to 8.7% ($p < 0.001$; study 2).

	Study 3: compared product A at 2 concentrations of AA, 624 and 1249 pmol/L (110 and 220 mg/L).<br><br>All infants received 4 labelled feeds, 2 feeds containing each isotope. 1 labelled feed/d was fed on 4 consecutive d.		dephytinised by the addition of phytase to the soy isolate (product B, 83% dephytinised; product D, 100% dephytinised).<br><br>Zn and AA were added. Within each study, 1 formula was labelled with 57Fe and the other with 58Fe.				Increasing the Fe:AA molar ratio from 1:2.1 to 1:4.2 in formula containing the native level of PA boosted mean Fe incorporation ($p < 0.05$) from 5.9 to 9.6% (study 3).<br><br>An inverse correlation ($p < 0.05$) was found between serum ferritin and Fe incorporation of both test meals in study 1 (A and B).<br><br>Fe incorporation from test meal A was found to be age correlated ($p < 0.05$).
**[40]**	**Aim:** To determine the effect of dietary PA on Zn homeostasis and to test the effect of age on maintaining Zn homeostasis with the changes of dietary PA in the Korean population.<br><br>**Design:** Interventional study<br><br>Subjects were studied consecutively for 3 mo in 2 metabolic periods (MP) in 2 different metabolic U.<br><br>During MP1 the women consumed a HP diet (P:Zn molar ratio 23) for 9 d.	Type of phytase: *Aspergillus* niger<br><br>Dose: 5000 U/g<br><br>Duration: 9 d	**Type of meal:** LP diet	N = 17<br><br>Healthy Adults	**Country:** Korea<br><br>**Setting:** Seoul National University	FAZ<br><br>EFZ	Plasma Zn concentrations were about 33% higher in the elderly than younger women in both metabolic periods.<br><br>A high PA diet increased total faecal Zn excretion and reduced the amount of total absorbed Zn in both young women and elderly women.<br><br>Endogenous faecal and urinary Zn losses were not reduced during the high PA metabolic

	After a 10 d wash-out period at home eating their usual diets, a LP diet (P:Zn molar ratio 10) was fed in MP2 for 9 d.<br><br>Phytase was added to selected foods in the HP diet to reduce the PA content of the meals in the LP period.						period.<br><br>It appears that in the short-term, higher intakes of PA mainly impacts intestinal Zn absorption rather than secretion or reabsorption.
**[41]**	**Aim:** The objective was to test whether children on a PA-reduced corn-soy diet have better Zn absorption and conservation than those on a standard HP diet.<br><br>**Design:** RCT<br><br>Randomly assigned a) standard diet, b) PA-reduced diet for 3-7 d.<br><br>The diet was corn-plus-soy porridge served 5/d.<br><br>PA-reduced diet: addition of commercial phytase enzyme, 1 g of flour/kg was added to cooled porridge. Identical menus for both diets, except the porridge PA reduction.<br><br>Supplement: Zn-free multivitamin daily.	**Type of phytase:** *Aspergillus* niger<br><br>**Dose:** Phytase enzyme (5000 U/g; BASF, Mount Olive, NJ)<br><br>**Duration**: 3-7 d	**Type of meal:** corn-plus-soy porridge	N = 23<br><br>Recovering children (from TB, minor trauma or elective orthopaedic procedure) and healthy siblings (3–13 y)	**Country**: Malawi<br><br>**Setting**: Hospital	FAZ<br><br>TAZ	For the recovering children, dietary PA reduction resulted in greater FAZ and TAZ.<br><br>In contrast, among the healthy children, dietary PA reduction had no effect on Zn absorption.
**[42]**	**Aim:** The objective of the study was to evaluate the impact of PA on Fe bioavailability from Fe-biofortified beans.<br><br>**Design:** RCT with Crossover Design	**Type of phytase:** *Aspergillus* niger	**Type of meal**: Beans (biofortified, control, 50% dephytinised or	N=22<br><br>Healthy Adults	**Country:** Rwanda<br><br>**Setting:** National	Fe absorption	PA strongly decreases Fe bioavailability from Fe-biofortified beans, and a high PA concentration limits the optimal effectiveness of bean Fe biofortification.

	Fe absorption from meals containing biofortified beans and control beans was measured with beans containing either their native PA concentration or with beans that were 50% dephytinised or >95% dephytinised.<br><br>The study was divided into 3 2-wk feeding periods, which were separated by 2-wk rest periods. In each feeding period 2 different bean meals were served (in total, 6 different bean meals). 3 test bean meals were prepared from control cream-striped (carioca) beans and 3 test meals were prepared from cream-striped (carioca) biofortified beans. The test meals contained either beans with their native PA concentration or beans that were ;50% or >95% dephytinised.	**Dose:** 8 PTU/g of beans<br><br>**Duration:** 3 2-wk feeding periods	>95% dephytinised).<br><br>The 50% dephytinised bean meals were prepared by mixing dephytinised and nondephytinised bean slurries in equal proportions.<br><br>Biofortified beans (8.8 mg Fe, 1320 mg PA/100 g) and control beans (5.4 mg Fe, 980 mg PA/100 g).		University of Rwanda		The Fe concentration of the cooked composite meals with biofortified beans was 54% higher than in the control meals. Fractional Fe absorption from the control bean meals was 9.2%, 30% higher than that from the biofortified bean meals ($P < 0.001$).<br><br>The quantity of Fe absorbed from the biofortified bean meals (406 mg) was 19% higher ($P < 0.05$) than that from the control meals. With 50% and >95% dephytinisation, Fe absorption from biofortified beans increased to 599 and 746 mg, respectively, which was 37% ($P < 0.005$) and 51% ($P < 0.0001$) compared to the control meals.

Abbreviations: PA: Phytic Acid, NS: Not specified, Fe: Iron, Zn: Zinc, Ca: Calcium, Cu: Copper, Mn: Manganese, Se: Selenium, P: Phosphorus, FTU or PTU: Phytase units, D: day, Wk: weeks, Mo: months, FFQ: Food Frequency Questionnaire, TB: Tuberculosis, CB: Control bread, TB: Teff bread, P1: Level 1 phytase, P2: Level 2 phytase, FFP: Single non-dephytinised fonio flour porridge, FWFP: Mixed dephytinised fonio-wheat porridge, T: Test product, R: Reference dose, RM: Reference meal, PP: Polyphenol compounds, MP: Metabolic periods, AA: Ascorbic acid, FAZ: Fractional absorption of Zn, EFZ: Endogenous faecal Zn excretion, LP: Low PA, HP: High PA, SCC: Serum concentration curve

References:

1. Herter-Aeberli I, Fischer MM, Egli IM, Zeder C, Zimmermann MB, Hurrell RF: **Addition of Whole Wheat Flour During Injera Fermentation Degrades Phytic Acid and Triples Iron Absorption from Fortified Tef in Young Women**. *J Nutr* 2020, **150**(10):2666-2672.
2. Zyba SJ, Wegmüller R, Woodhouse LR, Ceesay K, Prentice AM, Brown KH, Wessells KR: **Effect of exogenous phytase added to small-quantity lipid-based nutrient supplements (SQ-LNS) on the fractional and total absorption of zinc from a millet-based porridge consumed with SQ-LNS in young Gambian children: a randomized controlled trial**. *Am J Clin Nutr* 2019, **110**(6):1465-1475.
3. Monnard A, Moretti D, Zeder C, Steingötter A, Zimmermann MB: **The effect of lipids, a lipid-rich ready-to-use therapeutic food, or a phytase on iron absorption from maize-based meals fortified with micronutrient powders**. *Am J Clin Nutr* 2017, **105**(6):1521-1527.
4. Brnić M, Wegmüller R, Melse-Boonstra A, Stomph T, Zeder C, Tay FM, Hurrell RF: **Zinc Absorption by Adults Is Similar from Intrinsically Labeled Zinc-Biofortified Rice and from Rice Fortified with Labeled Zinc Sulfate**. *J Nutr* 2016, **146**(1):76-80.
5. Brnić M, Wegmüller R, Zeder C, Senti G, Hurrell RF: **Influence of phytase, EDTA, and polyphenols on zinc absorption in adults from porridges fortified with zinc sulfate or zinc oxide**. *J Nutr* 2014, **144**(9):1467-1473.
6. Cercamondi CI, Egli IM, Mitchikpe E, Tossou F, Hessou J, Zeder C, Hounhouigan JD, Hurrell RF: **Iron bioavailability from a lipid-based complementary food fortificant mixed with millet porridge can be optimized by adding phytase and ascorbic acid but not by using a mixture of ferrous sulfate and sodium iron EDTA**. *J Nutr* 2013, **143**(8):1233-1239.
7. Troesch B, van Stuijvenberg ME, Smuts CM, Kruger HS, Biebinger R, Hurrell RF, Baumgartner J, Zimmermann MB: **A micronutrient powder with low doses of highly absorbable iron and zinc reduces iron and zinc deficiency and improves weight-for-age Z-scores in South African children**. *J Nutr* 2011, **141**(2):237-242.
8. Troesch B, Egli I, Zeder C, Hurrell RF, de Pee S, Zimmermann MB: **Optimization of a phytase-containing micronutrient powder with low amounts of highly bioavailable iron for in-home fortification of complementary foods**. *Am J Clin Nutr* 2009, **89**(2):539-544.
9. Bach Kristensen M, Tetens I, Alstrup Jørgensen AB, Dal Thomsen A, Milman N, Hels O, Sandström B, Hansen M: **A decrease in iron status in young healthy women after long-term daily consumption of the recommended intake of fibre-rich wheat bread**. *Eur J Nutr* 2005, **44**(6):334-340.
10. Layrisse M, García-Casal MN, Solano L, Barón MA, Arguello F, Llovera D, Ramírez J, Leets I, Tropper E: **Iron bioavailability in humans from breakfasts enriched with iron bis-glycine chelate, phytates and polyphenols**. *J Nutr* 2000, **130**(9):2195-2199.
11. Sandberg AS, Hulthén LR, Türk M: **Dietary Aspergillus niger phytase increases iron absorption in humans**. *J Nutr* 1996, **126**(2):476-480.
12. Adams CL, Hambidge M, Raboy V, Dorsch JA, Sian L, Westcott JL, Krebs NF: **Zinc absorption from a low-phytic acid maize**. *Am J Clin Nutr* 2002, **76**(3):556-559.

13. Armah SM, Boy E, Chen D, Candal P, Reddy MB: **Regular Consumption of a High-Phytate Diet Reduces the Inhibitory Effect of Phytate on Nonheme-Iron Absorption in Women with Suboptimal Iron Stores**. *J Nutr* 2015, **145**(8):1735-1739.
14. Bohn T, Davidsson L, Walczyk T, Hurrell RF: **Phytic acid added to white-wheat bread inhibits fractional apparent magnesium absorption in humans**. *Am J Clin Nutr* 2004, **79**(3):418-423.
15. Brune M, Rossander-Hultén L, Hallberg L, Gleerup A, Sandberg AS: **Iron absorption from bread in humans: inhibiting effects of cereal fiber, phytate and inositol phosphates with different numbers of phosphate groups**. *J Nutr* 1992, **122**(3):442-449.
16. Brune M, Rossander L, Hallberg L: **Iron absorption: no intestinal adaptation to a high-phytate diet**. *Am J Clin Nutr* 1989, **49**(3):542-545.
17. Delimont NM, Nickel S: **Salivary cystatin SN is a factor predicting iron bioavailability after phytic acid rich meals in female participants**. *Int J Food Sci Nutr* 2021, **72**(4):559-568.
18. Egli I, Davidsson L, Zeder C, Walczyk T, Hurrell R: **Dephytinization of a complementary food based on wheat and soy increases zinc, but not copper, apparent absorption in adults**. *J Nutr* 2004, **134**(5):1077-1080.
19. Fredlund K, Isaksson M, Rossander-Hulthén L, Almgren A, Sandberg AS: **Absorption of zinc and retention of calcium: dose-dependent inhibition by phytate**. *J Trace Elem Med Biol* 2006, **20**(1):49-57.
20. Hambidge KM, Miller LV, Mazariegos M, Westcott J, Solomons NW, Raboy V, Kemp JF, Das A, Goco N, Hartwell T *et al*: **Upregulation of Zinc Absorption Matches Increases in Physiologic Requirements for Zinc in Women Consuming High- or Moderate-Phytate Diets during Late Pregnancy and Early Lactation**. *J Nutr* 2017, **147**(6):1079-1085.
21. Hambidge KM, Krebs NF, Westcott JL, Sian L, Miller LV, Peterson KL, Raboy V: **Absorption of calcium from tortilla meals prepared from low-phytate maize**. *Am J Clin Nutr* 2005, **82**(1):84-87.
22. Heaney RP, Weaver CM, Fitzsimmons ML: **Soybean phytate content: effect on calcium absorption**. *Am J Clin Nutr* 1991, **53**(3):745-747.
23. Hoppe M, Ross AB, Svelander C, Sandberg AS, Hulthén L: **Low-phytate wholegrain bread instead of high-phytate wholegrain bread in a total diet context did not improve iron status of healthy Swedish females: a 12-week, randomized, parallel-design intervention study**. *Eur J Nutr* 2019, **58**(2):853-864.
24. Hunt JR, Beiseigel JM: **Dietary calcium does not exacerbate phytate inhibition of zinc absorption by women from conventional diets**. *Am J Clin Nutr* 2009, **89**(3):839-843.
25. Lind T, Lönnerdal B, Persson LA, Stenlund H, Tennefors C, Hernell O: **Effects of weaning cereals with different phytate contents on hemoglobin, iron stores, and serum zinc: a randomized intervention in infants from 6 to 12 mo of age**. *Am J Clin Nutr* 2003, **78**(1):168-175.
26. Mazariegos M, Hambidge KM, Krebs NF, Westcott JE, Lei S, Grunwald GK, Campos R, Barahona B, Raboy V, Solomons NW: **Zinc absorption in Guatemalan schoolchildren fed normal or low-phytate maize**. *Am J Clin Nutr* 2006, **83**(1):59-64.

27. Petry N, Rohner F, Gahutu JB, Campion B, Boy E, Tugirimana PL, Zimmerman MB, Zwahlen C, Wirth JP, Moretti D: **In Rwandese Women with Low Iron Status, Iron Absorption from Low-Phytic Acid Beans and Biofortified Beans Is Comparable, but Low-Phytic Acid Beans Cause Adverse Gastrointestinal Symptoms**. *J Nutr* 2016, **146**(5):970-975.
28. Petry N, Egli I, Campion B, Nielsen E, Hurrell R: **Genetic reduction of phytate in common bean (Phaseolus vulgaris L.) seeds increases iron absorption in young women**. *J Nutr* 2013, **143**(8):1219-1224.
29. Bokhari F, Derbyshire E, Li W, Brennan CS, Stojceska V: **A study to establish whether food-based approaches can improve serum iron levels in child-bearing aged women**. *J Hum Nutr Diet* 2012, **25**(1):95-100.
30. Davidsson L, Ziegler EE, Kastenmayer P, van Dael P, Barclay D: **Dephytinisation of soyabean protein isolate with low native phytic acid content has limited impact on mineral and trace element absorption in healthy infants**. *Br J Nutr* 2004, **91**(2):287-294.
31. Davidsson L, Galan P, Cherouvrier F, Kastenmayer P, Juillerat MA, Hercberg S, Hurrell RF: **Bioavailability in infants of iron from infant cereals: effect of dephytinization**. *Am J Clin Nutr* 1997, **65**(4):916-920.
32. Davidsson L, Almgren A, Juillerat MA, Hurrell RF: **Manganese absorption in humans: the effect of phytic acid and ascorbic acid in soy formula**. *Am J Clin Nutr* 1995, **62**(5):984-987.
33. Hurrell RF, Reddy MB, Juillerat MA, Cook JD: **Degradation of phytic acid in cereal porridges improves iron absorption by human subjects**. *Am J Clin Nutr* 2003, **77**(5):1213-1219.
34. Koréissi-Dembélé Y, Fanou-Fogny N, Moretti D, Schuth S, Dossa RA, Egli I, Zimmermann MB, Brouwer ID: **Dephytinisation with intrinsic wheat phytase and iron fortification significantly increase iron absorption from fonio (Digitaria exilis) meals in West African women**. *PLoS One* 2013, **8**(10):e70613.
35. Zhang H, Onning G, Oste R, Gramatkovski E, Hulthén L: **Improved iron bioavailability in an oat-based beverage: the combined effect of citric acid addition, dephytinization and iron supplementation**. *Eur J Nutr* 2007, **46**(2):95-102.
36. Manary MJ, Krebs NF, Gibson RS, Broadhead RL, Hambidge KM: **Community-based dietary phytate reduction and its effect on iron status in Malawian children**. *Ann Trop Paediatr* 2002, **22**(2):133-136.
37. Petry N, Egli I, Zeder C, Walczyk T, Hurrell R: **Polyphenols and phytic acid contribute to the low iron bioavailability from common beans in young women**. *J Nutr* 2010, **140**(11):1977-1982.
38. Couzy F, Mansourian R, Labate A, Guinchard S, Montagne DH, Dirren H: **Effect of dietary phytic acid on zinc absorption in the healthy elderly, as assessed by serum concentration curve tests**. *Br J Nutr* 1998, **80**(2):177-182.
39. Davidsson L, Galan P, Kastenmayer P, Cherouvrier F, Juillerat MA, Hercberg S, Hurrell RF: **Iron bioavailability studied in infants: the influence of phytic acid and ascorbic acid in infant formulas based on soy isolate**. *Pediatr Res* 1994, **36**(6):816-822.
40. Kim J, Paik HY, Joung H, Woodhouse LR, Li S, King JC: **Effect of dietary phytate on zinc homeostasis in young and elderly Korean women**. *J Am Coll Nutr* 2007, **26**(1):1-9.

41. Manary MJ, Hotz C, Krebs NF, Gibson RS, Westcott JE, Arnold T, Broadhead RL, Hambidge KM: **Dietary phytate reduction improves zinc absorption in Malawian children recovering from tuberculosis but not in well children**. *J Nutr* 2000, **130**(12):2959-2964.
42. Petry N, Egli I, Gahutu JB, Tugirimana PL, Boy E, Hurrell R: **Phytic acid concentration influences iron bioavailability from biofortified beans in Rwandese women with low iron status**. *J Nutr* 2014, **144**(11):1681-1687.
